# Barriers to Diabetes Self-management Education Programs in Underserved Rural Arkansas: Implications for Program Evaluation

**Published:** 2005-12-15

**Authors:** Appathurai Balamurugan, Mark Rivera, Leonard Jack, Sharon Morris, Kristen Allen

**Affiliations:** Arkansas Department of Health and Human Services; Dr Balamarugan is also an assistant professor in the Fay W. Boozman College of Public Health at the University of Arkansas for Medical Sciences, Little Rock, Ark; Applied Behavioral Research, Epidemiology, Surveillance, and Evaluation (ABRESE), Centers for Disease Control and Prevention, Division of Diabetes Translation, Program Development Branch, Atlanta, Ga; Applied Behavioral Research, Epidemiology, Surveillance, and Evaluation (ABRESE), Centers for Disease Control and Prevention, Division of Diabetes Translation, Program Development Branch, Atlanta, Ga; Applied Behavioral Research, Epidemiology, Surveillance, and Evaluation (ABRESE), Centers for Disease Control and Prevention, Division of Diabetes Translation, Program Development Branch, Atlanta, Ga; Arkansas Diabetes Prevention and Control Program, Little Rock, Ark

## Abstract

**Background:**

Diabetes prevalence has reached epidemic proportions. Diabetes self-management education (DSME) has been shown to improve preventive care practices and clinical outcomes. In this study, we discuss the barriers faced during the implementation of DSME programs in medically underserved rural areas of Arkansas.

**Context:**

Arkansas is a rural state, with most southeastern counties experiencing a shortage of health care professionals. The Arkansas Diabetes Prevention and Control Program and its partners established 12 DSME programs in underserved counties with a high prevalence of diabetes.

**Methods:**

DSME programs were delivered by a registered nurse and a dietitian who provided 10 to 13 hours of education to each program participant. Baseline, 6-month, and year-end data were collected on preventive care practices, such as daily blood glucose monitoring, foot examination, systolic and diastolic blood pressure, and glycosylated hemoglobin level, among the participants in newly established DSME programs.

**Consequences:**

Of the 12 DSME programs established, 11 received American Diabetes Association recognition. The number of participants in the DSME programs increased 138% in 1 year, from 308 in February 2003 to 734 in March 2004. Preventive care practices improved: daily blood glucose monitoring increased from 56% to 67% of participants, and daily foot examinations increased from 63% to 84% of participants. Glycosylated hemoglobin decreased by an average of 0.5 units per participant who completed the program. However, many anticipated and a few unanticipated barriers during the implementation of the program could not be overcome because of the lack of an evaluation plan.

**Interpretation:**

Although results point to potential benefits of preventive care practices among DSME participants, interpretation of findings was limited by sample size. Sample size limitations are traced to barriers to assessing program outcome. Program evaluation should be integrated into the planning phase to ensure adequate measures of program effectiveness.

## Background

Diabetes prevalence has reached epidemic proportions in the United States. In 2002, 18 million people were estimated to have diabetes (1). The direct medical and indirect expenditures attributable to diabetes were estimated at $132 billion in 2002 (2). Future projections indicate that diabetes prevalence will continue to increase, expenditures will remain high, and diabetes will continue to be a serious health concern (1). Establishing the efficacy and effectiveness of disease management and education interventions that target health care providers, patients, families, and communities is critically important.

A systematic review of published studies addressing the effectiveness of population-based diabetes-related interventions recommends diabetes self-management education (DSME) (3). DSME empowers people to manage diabetes through education about nutrition, medication and insulin therapy, stress management, and preventive foot and eye care (4). DSME has been shown to be effective in community settings (5).

Although few studies have examined the challenges and barriers associated with establishing DSME programs in underserved areas (6,7), issues such as accessibility to quality health care in underserved areas have been well documented (8). Studies of barriers to quality health care have mostly addressed patient-level factors such as transportation and financial issues; system-level factors affecting program implementation in underserved rural areas are seldom mentioned. Incorporating formative evaluation during DSME program conception is one way to identify and overcome some of the barriers faced during program implementation (9). In this study, we discuss the barriers faced during the implementation of DSME programs in medically underserved rural areas of Arkansas. We also discuss measures taken to overcome them and lessons learned from not having an evaluation plan.

## Context

Arkansas is a rural state, with most counties in southeast Arkansas designated by the Health Services and Resources Administration as areas with a shortage of health professionals. Diabetes prevalence in Arkansas has been higher than the national average for the past decade, with 7.9% of Arkansans aged 18 years and older diagnosed with diabetes in 2002 (10). Costs for diabetes-related hospitalizations in Arkansas in 2001 were estimated to be $55 million.

**Figure 1:**
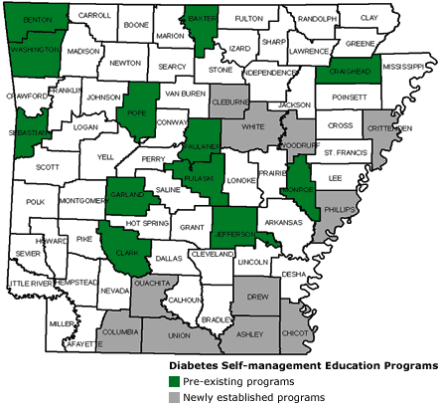
Distribution of pre-existing and newly established diabetes self-management education (DSME) programs recognized by the American Diabetes Association in Arkansas, by county.

**Figure 2:**
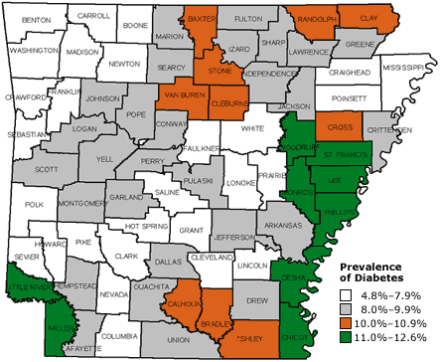
Prevalence of diabetes in Arkansas, by county, 2002. Source: Behavioral Risk Factor Surveillance System.

DSME reduces diabetes complications as well as associated costs (11). In 2001, only 42% of Arkansans diagnosed with diabetes had ever participated in a DSME program (10). This low percentage may have partly resulted from DSME programs being located primarily within central and northwestern counties of the state (Figure 1), whereas the prevalence of diabetes is disproportionately higher in southeastern counties (i.e., counties within the Mississippi Delta region) (Figure 2). The southeastern counties are more impoverished, more rural, and have poorer health care infrastructure than other counties. Also, most of these counties have a higher proportion of racial and ethnic minorities (up to 50%), predominantly African Americans, than the state overall (16%).

**Table:** 

**Table:** 

The Arkansas Diabetes Prevention and Control Program (ADPCP) assembled a coalition of public and private partners to establish DSME programs in counties with a high prevalence of diabetes. Particular attention was paid to counties with no DSME programs. The ADPCP used this opportunity to help Arkansas reach the *Healthy People 2010* target to provide diabetes education to 60% of people in the state diagnosed with diabetes (12). The ADPCP also intended to assess DSME program effectiveness in an effort to improve preventive care practices and clinical outcomes.

## Methods

### The ADPCP coalition

In fall 2001, the ADPCP formed a coalition consisting of public entities including the Department of Human Services, the Arkansas Foundation for Medical Care, Health Information Design, the American Diabetes Association (ADA), and the Arkansas Minority Health Commission. The coalition also included private entities (e.g., Eli Lilly and Company). The coalition's goal was to establish 12 high-quality DSME programs in underserved rural areas with a disproportionately high prevalence of diabetes. Objectives included identifying and recruiting hospitals and clinics interested in establishing DSME programs by February 2003, assisting with the resources required to establish DSME programs, assisting the DSME programs in obtaining ADA recognition by June 2003, assisting program instructors to become certified diabetes educators, and assessing program effectiveness at the end of 1 year of recruitment of all DSME programs. The coalition members identified their roles and responsibilities and worked together in making decisions for recruiting clinics, providing assistance with resources, and evaluating the intervention.

### Recruitment of DSME sites

The coalition identified underserved areas across the state as potential sites for DSME programs and assessed the existing infrastructure in those areas. Three certified diabetes educators were hired to assist with site recruitment and program implementation. Key hospital or clinic staff members (e.g., chief executive officers, medical directors) in underserved areas were contacted by telephone to assess their interest in establishing the DSME program. Coalition members and diabetes educators then conducted a 1-day visit with key personnel at each site expressing interest. They discussed the benefits of DSME, resources that could be provided to establish a program, details of the ADA-recognition application process, and reimbursement benefits of ADA recognition. The coalition provided sample educational tools and additional information through follow-up telephone calls. Based on these solicitations, the first 12 clinics that expressed interest were recruited to establish a DSME program. The clinics signed a memorandum of agreement for their roles and responsibilities, which included patient enrollment, patient education (DSME), and data collection, in return for the resources made available to them by the coalition. The recruitment phase began in January 2002, and 12 DSME programs were established by February 2003. DSME programs identified and enrolled people with diabetes through local physicians, pharmacies, and grocery stores.

### Resources provided to DSME sites

Each site that established a DSME program received resources, including the ADA program manual (*Life with Diabetes*); a copy of *Core Curriculum for Diabetes Education* (13); a license and payment of monthly fees for Dia-Trac, an online data collection system (Control Diabetes Services, Plano, Tex); a glycosylated hemoglobin (HbA1c) analyzer; professional consultation provided by three certified diabetes educators; continuing education credits provided through workshops arranged by the coalition; and reimbursement of cost for ADA recognition. The diabetes educators also assisted program staff to become certified diabetes educators. Funding was made available by Eli Lilly and Company. This financial support was exclusively intended and used for public health promotion and not to promote or influence the use of any Eli Lilly product.

### Intervention

DSME was provided to program participants by a registered nurse and a registered dietitian who followed the ADA core curriculum (13). Following the ADA curriculum helped ensure provision of quality diabetes education. After a 1-hour assessment of their educational needs, participants received 10 hours of diabetes education and 3 hours of medical nutrition therapy. Diabetes education was divided into three visits: an initial visit occurring shortly after the initial education assessment, a second at 6 months, and the third 1 year after program entry.

The diabetes education for each visit was provided in a group session. The curriculum addressed 10 content areas: the diabetes disease process; nutrition; physical activity; medications; monitoring and using test results; acute complications; chronic complications; goal setting and problem solving; psychosocial adjustment; and preconception care, pregnancy, and gestational diabetes (13). The diabetes education sessions were tailored to fit participants' needs. During each visit, educators gathered information from participants through questionnaires, including questions on demographics, self-care skills, and preventive care practices. The program staff members entered the data from the questionnaire into the Dia-trac data collection system. Control Diabetes Services was responsible for obtaining written informed consent from all patients and protecting the confidentiality of the data. The senior epidemiologist for the Arkansas Department of Health obtained the aggregate data from Control Diabetes Services with all identities removed.

## Consequences

### Program participants

The number of participants enrolled in the 12 DSME programs increased from 308 in February 2003 to 734 in March 2004. Of these 734 participants, 93% had type 2 diabetes. More than 75% were aged 45 years or older; 69% were white, and 30% were African American. More than 50% did not have a college degree.

Of the 319 participants due for the 1-year visit, only 20% (65) completed the 13 hours of diabetes education. Data were collected for 43 of these 65 participants on daily blood glucose monitoring, daily foot examination, and systolic and diastolic blood pressure. HbA1c level was obtained for 27 participants. There was some evidence of improvement in daily blood glucose monitoring, daily foot examination, systolic and diastolic blood pressure, and HbA1c levels (Table 1). These changes were not statistically significant, except for daily foot examination at baseline compared with 6-month follow-up (*P* = .03). The average HbA1c value for participants who completed the DSME program decreased from 8.15 at baseline to 7.65 at year end, a decrease of 0.5 units.

### Barriers to program implementation

Barriers to program implementation were frequently identified through informal discussions among coalition members and DSME program staff. Patient-level barriers were identified and reported to the coalition by the DSME program staff. The coalition held a monthly teleconference with the DSME program staff to discuss progress and barriers experienced at both program and patient levels. During these calls, approaches to overcoming some of the barriers were proposed. The coalition members worked on applying solutions to the program implementation barriers. DSME program staff members worked to address patient-level barriers within their own clinics. There were anticipated and unanticipated barriers to implementation at both the patient and program levels. Table 2 provides a summary of strategies used to minimize or eliminate anticipated and unanticipated barriers.

**Anticipated barriers**

At the program level, anticipated barriers centered on staffing and reimbursement for DSME. To obtain ADA recognition, the program needed at least one registered nurse and one registered dietician. Arkansas is a predominantly rural state, and more than half (58%) of its population lives in areas having a shortage of health professionals. Recruiting health professionals, particularly registered dietitians, was a challenge. Some DSME programs shared a registered dietitian to fulfill the ADA requirement.

Reimbursement constraints took a number of forms. Insurance reimbursement only took place after ADA recognition of a DSME program, which did not occur until 6 months into the program. Although the coalition was not able to provide financial assistance during this period, the resources provided to DSME programs helped to overcome this barrier. A related barrier was that although there was no formal pre-existing DSME program in the participating counties, most counties included diabetes education as a subcomponent of their broader health care services. Rural health centers were not always reimbursed because diabetes education was considered a service already available. These rural health centers perceived the DSME program as contributing beyond their current services, so they applied for grants to cover program costs. This was one approach used by DSME programs to secure additional funds.

Anticipated patient-level barriers included transportation, literacy, and reimbursement. Patients with no means of transportation needed to travel long distances to reach a DSME program site. To address this barrier, some DSME programs provided transportation by hospital vehicles; others coordinated transportation through local churches. Some patients had very little formal education, which presented a substantial barrier to understanding key DSME messages. In response, program staff members assisted patients by reading the materials to them. Medicaid members were not reimbursed for diabetes education. This barrier was anticipated, but the coalition was not able to overcome it. Because Medicaid members were asked to pay for DSME at their own expense, many dropped out of the program.

**Unanticipated barriers**

Unanticipated barriers included a lack of consistent data collection processes among DSME sites and participant retention. DSME programs were asked to enter participant information into the data collection system regularly, but this was not consistently done. Some program staff members said they lacked the resources (people or time) for data entry. Because the coalition could not assist with data entry and staff members did not understand the significance of gaps in data collection, the problem remained unsolved. Motivation to collect data was further decreased once sites received ADA recognition.

Participant retention posed a challenge partly as a result of environmental factors associated with rural health settings. The coalition's intent was to establish DSME programs in underserved areas of high diabetes prevalence where DSME programs would not have been available otherwise. However, the participation rate fell to 34% at the 6-month visit and 20% at the end-of-year visit. Some, but not all, program staff members reminded participants of their impending visits by postcard or telephone call.

### Evaluation results and lessons learned

The ADPCP hired an epidemiologist during fall 2002 after the program had formally begun. The epidemiologist engaged key stakeholders and DSME program staff in spring 2003 to plan and implement a program evaluation. Although integrating evaluation early in the program planning process can be very helpful, this is often not done for fear that evaluation will be seen as punitive, exclusionary, and adversarial (10). This was true in the present study. There was also no logic model developed by the coalition, although process measures were put in place to capture program implementation at each site. Information gleaned from these measures will be used to shape future DSME programs and to develop a DSME program logic model that may foster a clearer understanding of the barriers faced by these programs in rural Arkansas and their relationship to program outcomes.

## Interpretation

### Progress toward program goals and objectives

Key evaluation issues chosen to assess DSME program outcomes included whether 1) the coalition was able to establish 12 DSME programs in rural underserved counties in Arkansas, 2) the DSME program fostered progress toward achieving the *Healthy People 2010* target to provide diabetes education to 60% of people with diabetes, and 3) the DSME program fostered preventive care practices.

The coalition met its goal of establishing the 12 DSME programs in underserved counties. Figure 1 shows the location of pre-existing and newly established DSME programs. Of the 12 DSME programs, 11 met the minimum participation and 6-month follow-up requirements and obtained ADA recognition. By the 6-month follow-up, DSME programs were required to have 1) a minimum of 20 patients enrolled and 2) a continuous quality improvement (CQI) measure for patients. All 12 DSME programs collected HbA1c results as a CQI measure for ADA recognition. One DSME program did not obtain ADA recognition during the time frame because it had fewer than 20 patients enrolled in the program. One DSME program staff member became a certified diabetes educator, and two staff members from other DSME programs are preparing to take the certification examination.

The number of people receiving diabetes education in Arkansas more than doubled from February 2003 to March 2004. This increase highlights success in addressing diabetes education among the most hard-to-reach populations in the state. Although recruitment efforts for the DSME program had some success, the lack of a unified effort to retain participants, along with reimbursement-related barriers, may have contributed to high rates of attrition.

Key stakeholders, including coalition members and DSME program staff, were given the evaluation results. The coalition understood the weaknesses of the follow-up and realized that evaluation should have been incorporated early in program planning. If an evaluation planning process had been incorporated into the early coalition meetings, it may have led to the identification of key barriers and resulted in changes to program content, resources, and timeline. These changes may have, in turn, increased program effectiveness and usefulness of evaluation findings. Another potential limitation is that clinics self-selected to establish the DSME programs. This may limit the generalizability of findings because participating clinics may not be representative of clinics in rural Arkansas.

### New DSME sites and program improvements

The ADPCP and its coalition members plan to implement six more DSME program sites in underserved rural Arkansas counties by spring 2006. For that purpose, coalition and DSME site staffs will incorporate evaluation planning before the new sites are fully implemented. Developing formative and impact evaluation plans prior to program implementation helps to ensure an evaluation provides useful information. Impact evaluations are used to determine the degree to which a program has led to desired changes and may also have implications for future programs. The coalition will consider which evaluation data are needed from each site to enable a comprehensive assessment of program goals for utility, feasibility, propriety, and accuracy (14).

Program and evaluation efforts for the six new sites will include a review of the evaluation findings of other similar DSME programs to determine how best to address attrition, data consistency, and other key barriers. For example, studies examined attrition rates for diabetes education programs that included a follow-up component (6); attrition rates in these studies ranged from 0% to 79%. These studies showed that attrition may be due to participant, researcher, study, or environmental factors. Attrition rates were found to decrease when participant factors such as motivation, values, and beliefs are encouraged and certain program outreach methods are used (15).

Establishing the 12 DSME programs in underserved rural areas of Arkansas provided important lessons about the importance of an evaluation plan. The authors view the development of an evaluation plan as a necessary and valuable initial step toward better addressing the educational needs of people diagnosed with diabetes in rural Arkansas.

Even in the face of serious resource challenges, the coalition attempted to address both anticipated and unanticipated barriers. Findings from this program evaluation will affect the establishment of future DSME sites in rural Arkansas. Particular attention will be given to an evaluation plan that embraces fiscal, human, and environmental factors that affect program planning, implementation, and sustainability. Findings from this evaluation may prove useful to others working in medically underserved rural communities throughout the United States.

## Figures and Tables

**Table 1:** Comparison of Data at Baseline, 6 Months, and 1 Year on Selected Clinical Measures for Participants in Diabetes Self-management Education (DSME) Programs, Arkansas, February 2003–March 2004^a^

**Diabetes Management Measure**	**No. of Participants**	**Baseline, % **	**6 Months, %**	**1 Year, % **
Daily blood glucose monitoring	43	56	70	67
Daily foot examination	43	63	91	84
Systolic blood pressure <130 mm Hg	43	44	36	50
Diastolic blood pressure <80 mm Hg	43	44	55	64
Hemoglobin A1c <7	27	19	30	30

a Increase among participants in daily foot examinations at 6 months was the only statistically significant change (McNemar test, *P* = .03).

**Table 2:** Anticipated and Unanticipated Barriers Faced in Establishing Diabetes Self-management Education (DSME) Programs in Underserved Areas, Arkansas, 2003–2004

**Barriers**	**Anticipated or Unanticipated**	**How Barrier Was Overcome**	**Lessons Learned**

**Program level**			

Recruitment of program sites	Anticipated	Coalition members provided hands-on training and technical assistance	Cost-effectiveness data are needed to increase buy-in among those interested and as a marketing tool to promote significance of DSME
Financial constraint	Anticipated	Coalition assisted with resources	Arkansas Diabetes Program should look for funds to sustain existing programs and to establish new programs
Insurance reimbursement to health centers	Anticipated	Funds were met through grants	Coalition is exploring opportunities for bridging gaps in funding
Shortage of registered dietitians	Anticipated	Programs shared their dietician	Shortage of registered dietitians must be addressed
Data collection	Unanticipated	Barrier could not be overcome	Evaluation plan and involvement of all stakeholders are essential during planning phase of program

**Patient level**			

Transportation	Anticipated	Transportation was provided from hospital or church	Relationship with local community organizations should be established
Literacy levels	Anticipated	Staff members assisted with reading and interpreting materials	Culturally and linguistically appropriate materials should be used
Reimbursement to Medicaid recipients	Anticipated	Barrier could not be overcome	Reimbursement issues negatively affected program retention
Retention	Unanticipated	Participants received postcard and telephone reminders from some DSME staff members	No unified effort to retain participants was made, possibly because of lack of evaluation plan
